# Supplementary material for: SARS-CoV-2 spike antigen-specific B cell and antibody responses in pre-vaccination period COVID-19 convalescent males and females with or without post-covid condition
Source: Front Immunol. 2023 Sep 21;14:1223936. doi: 10.3389/fimmu.2023.1223936 (PMC10551145; doi:10.3389/fimmu.2023.1223936)
Supplement: Supplementary file 1 [file DataSheet_1.pdf]

Supplementary Table S1. Antibodies and reagents used for flow cytometry.

Reagent	Source	Cat. #	Clone
Anti-human CD3 - AF700	BioLegend	300424	UCHT1
Anti-human CD14 - AF700	BioLegend	367114	63D3
Anti-human CD56 - AF700	BioLegend	318316	HCD56
Anti-human CD19 - APC-Cy7	BioLegend	302218	HIB19
Anti-human CD27 - BV605	BioLegend	302830	O323
Anti-human CD20 - PE-Cy7	BioLegend	302312	2H7
Anti-human IgD - BV785	BioLegend	348242	IA6-2
RBD D614G* -biotin	AcroBiosystems	SPD-C82E8	
RBD Omicron -biotin	AcroBiosystems	SPD-C82E4	
Streptavidin - APC	BioLegend	405207	
Streptavidin - FITC	BioLegend	405202	
DRAQ7 live/dead stain	BioLegend	424001	

* RBD D614G refers to the strain that was in circulation in 2020.

Supplementary Table S2. Materials used for ELISA

Material	Source	Cat #
96-well high-binding microtiter plate	Greiner Bio-One	655061
RBD D614G (ELISA)	AcroBiosystems	SPD-C52H3
RBD Omicron (ELISA)	AcroBiosystems	SPD-C522e
Spike Protein	NRC	SMT1-1
Nucleocapsid Protein	NRC	NCAP-1
Anti-human IgG	Sigma	B3773
Anti-human IgA	Sigma	SAB3701227
Anti-human IgG3	Sigma	B3523
Streptavidine-Peroxidase	Sigma	18-152
HRP-IgA Conjugate (for N protein)	Sigma	SAB3701236
HRP-IgG Conjugate (for N protein)	Sigma	A0170
TMB	Sigma	ES022

Supplementary Table S3. Plasma dilutions used for ELISA

Antibody specificity	Plasma dilution
Anti-D614G IgG RBD	1:1000
Anti-Omi IgG RBD	1:500
Anti-Spike IgG	1:1000
Anti-Spike, RBD IgG3	1:100
Anti-Spike, RBD IgA	1:500
Anti-N IgG	1:1000
Anti-N IgA	1:500 (3 months post-infection)
Anti-N IgA	1:1000 (1 month post-infection)
