# Supplementary material for: SARS-CoV-2 spike antigen-specific B cell and antibody responses in pre-vaccination period COVID-19 convalescent males and females with or without post-covid condition
Source: Front Immunol. 2023 Sep 21;14:1223936. doi: 10.3389/fimmu.2023.1223936 (PMC10551145; doi:10.3389/fimmu.2023.1223936)
Supplement: Supplementary file 2 [file DataSheet_2.pdf]

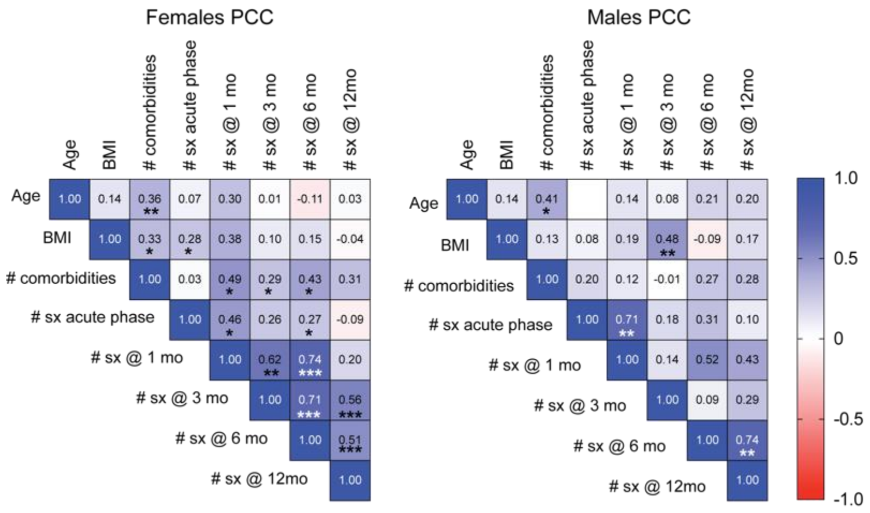


**Supplementary Figure S1. Correlation between clinical parameters of acute disease and PCC in females and males.**

Correlation matrix was generated between age, co-morbidities and the number of symptoms at the acute phase and at 1, 3, 6 or 12-months post-infection in convalescent COVID-19 females and males with PCC. The numbers in the squares indicate the Spearman coefficient value. Asterisks indicate the *p* values: * p<0.05; ** p<0.01; *** p<0.001. Actual *p* values are given in Supplementary Table S4. # sx- number of symptoms at the indicated months post-infection.

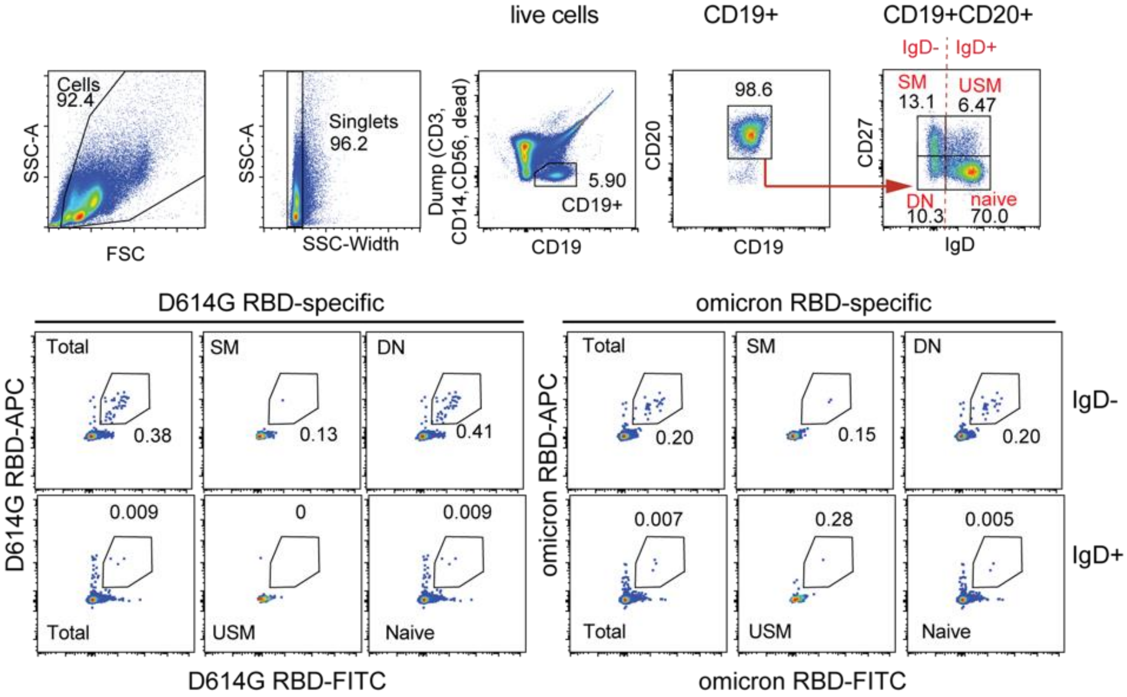

**Supplementary Figure S2. Gating strategy to quantify RBD-specific B cells.**

PBMCs were labelled with B cell markers (CD19, CD20, CD27 and IgD), AF-700 conjugated antibodies for T cells, monocytes and NK (dump channel), D614G-RBD and Omicron-RBD. Data was acquired with Cytoflex-30 cytometer and analyzed by FlowJo v10.3. a) $CD19^{+}CD20^{+}$ B lymphocytes were selected from the singlets after dumping T cells, NK cells and monocytes. CD27 and IgD expression within the B cells defined four subpopulations: naïve ($CD27^{-}IgD^{+}$), unswitched memory (USM; $CD27^{+}IgD^{+}$) switched memory (SM; $CD27^{+}IgD^{-}$), and double negative (DN; $CD27^{-}IgD^{-}$) B cells. Within these B cell subpopulations and total B cells the frequencies of D614G-RBD and Omicron-RBD reactive cells were estimated.

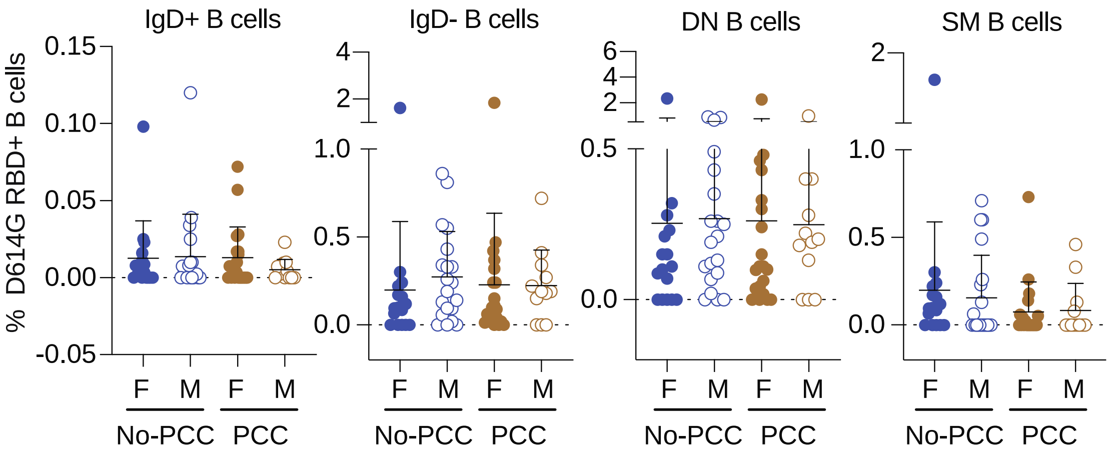


**Supplementary Figure S3: D614G-RBD specific B cell frequencies in convalescent PCC and No-PCC groups at 3 months post-infection.**

Data presented in Fig. 2 were segregated by sex and compared. No significant differences were observed between females and males in the frequencies of D614G RBD reactive B cell subsets as evaluated by Mann Whitney's test.

**Anti-RBD omicron**

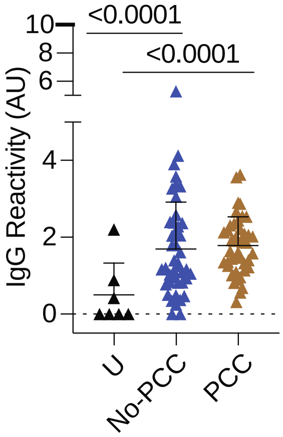


**Supplementary Figure S4. Anti- omicron RBD specific IgG responses in uninfected and convalescent individuals with or without PCC.**

Plasma samples were collected during routine clinical visit at 3 months post PCR-positive diagnosis. Anti-omicron IgG responses in uninfected (U), No-PCC and PCC groups were determined by ELISA. The groups were compared by Mann Whitney's test.

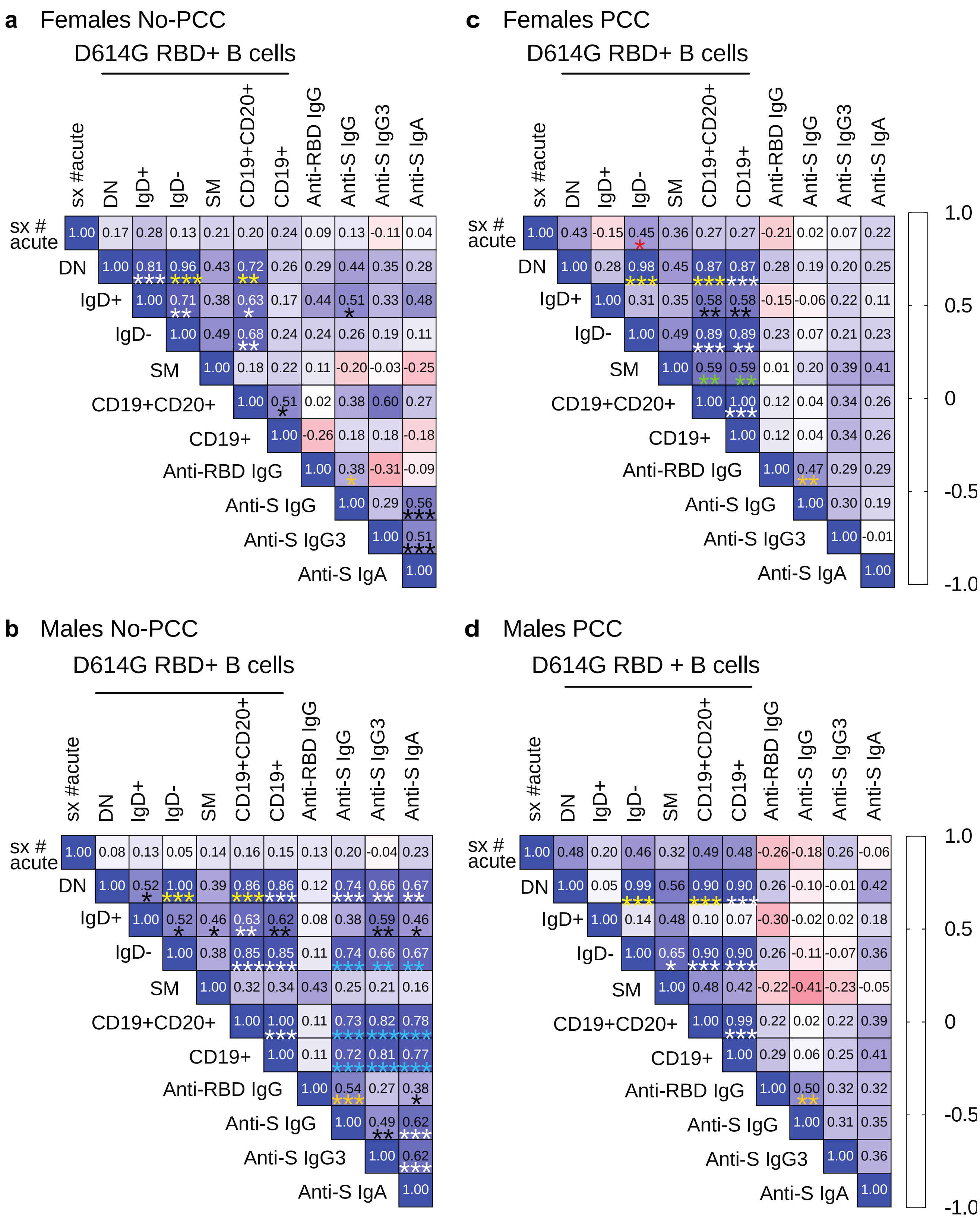
a Females No-PCC
D614G RBD+ B cells
c Females PCC
D614G RBD+ B cells
b Males No-PCC
D614G RBD+ B cells
d Males PCC
D614G RBD + B cells
sx #acute
DN
IgD+
IgD-
SM
CD19+CD20+
CD19+
Anti-RBD IgG
Anti-S IgG
Anti-S IgG3
Anti-S IgA
1.0
0.5
0
-0.5
-1.0

**Supplementary Figure S5. Correlation between SARS-CoV-2 Spike protein-specific B cell reactivities and antibody responses in PCC and No-PCC groups among males and females.** Correlation matrices were generated for the indicated parameters for the convalescent groups with or without PCC at 3 months post-infection. Nonparametric Spearman's correlation coefficient values (numbers) and their significance (asterisks) are indicated. * $p<0.05$, ** $p<0.01$, *** $p<0.001$. Asterisks are color coded to as visual aids to denote specific comparisons described in the text. The white and black numbers and asterisks are only used to contrast with the background color. The number of samples and exact *p* values are shown in the **Supplementary Table S5**.

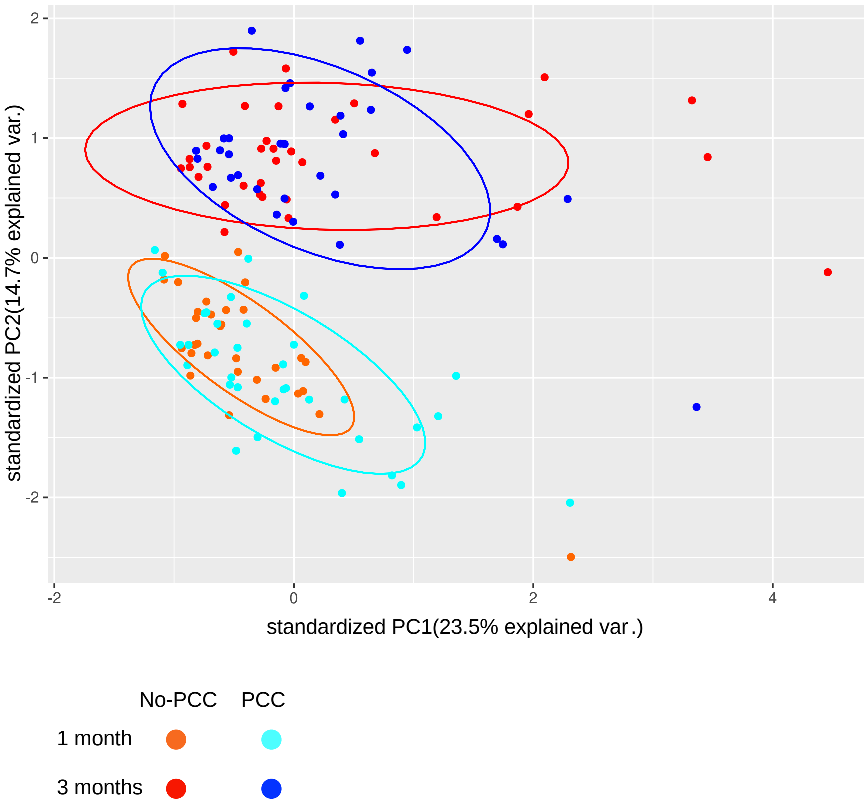


**Supplementary Figure S6. PCA analyses of clinical parameters, B cell reactivities and antibody responses to SARS-CoV-2 antigens in PCC and No-PCC groups at 1 and 3 months post-infection.**

The PCA plot was generated using the R 4.1.2 (https://cran.r-project.org/) ggbiplot package (https://rdocumentation.org/packages/ggbiplot/versions/0.55). Immune parameters for which more than 50% of the samples had values at both 1- and 3-month timepoints were utilized. These parameters included sex, number of symptoms at the acute infection timepoint, BMI, anti-D614G RBD specific B cell subsets ($CD19^+$, $CD19^+CD20^+$, $IgD^+$, $IgD^-$, naïve, DN, SM and USM), anti-D614G RBD IgG, anti-spike IgG, anti-spike IgG3, anti-spike IgA, anti-nucleocapsid IgG and anti-nucleocapsid IgA. Excluded from the data were data points with no values as well as those obtained after vaccination. PC1 and PC2 were plotted and a normal data ellipse for each group was generated.

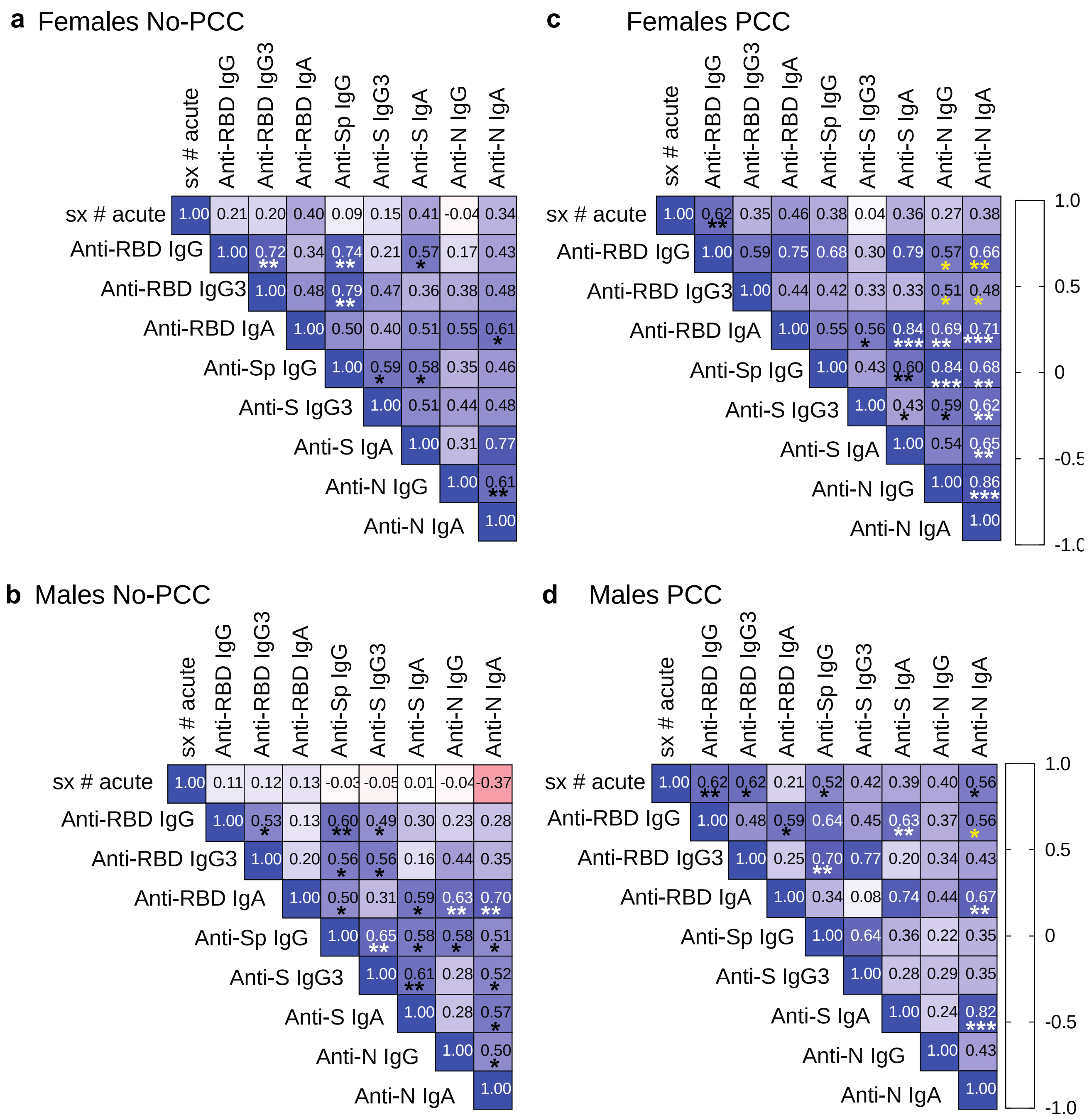


**Supplementary Figure S7. Correlation matrix for anti-RBD, spike and nucleocapsid antibody responses in PCC and No-PCC groups at 1-month post-infection.**

Correlation matrix for antibody responses at 1-month post infection in (**a**) females No-PCC, (**b**) males No-PCC, (**c**) females PCC and (**d**) males PCC groups. RBD refers to D614G-RBD. The numbers in the squares indicate the Spearman coefficient value. The *p* values are indicated in the figure as asterisks (*

*p*<0.05, ** *p*<0.01, *** *p*<0.001) and are given in **Supplementary Table S6.** Due to the limited quantity of samples in which B cell reactivities were measured, these parameters were not included in the correlation matrix and are given in **Supplementary Fig. S8**.

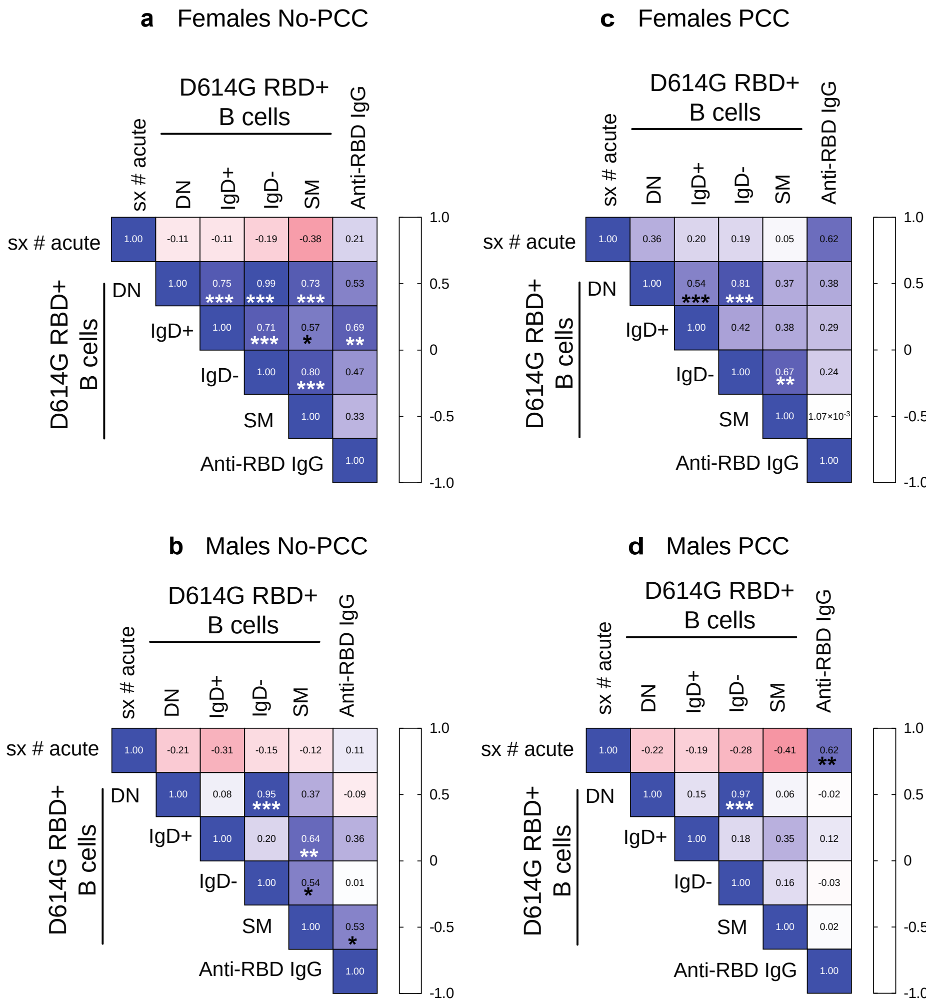


**Supplementary Figure S8.** Correlation matrix for B cell responses at 1- month post infection in females and males with and without PCC. The numbers in the squares indicate the Spearman coefficient value. The *p* values are indicated in the figure as asterisks ( * *p*<0.05, ** *p*<0.01, *** *p*<0.001) and are given in **Supplementary Table S6**. For simplicity they are indicated in the figure with asterisks.

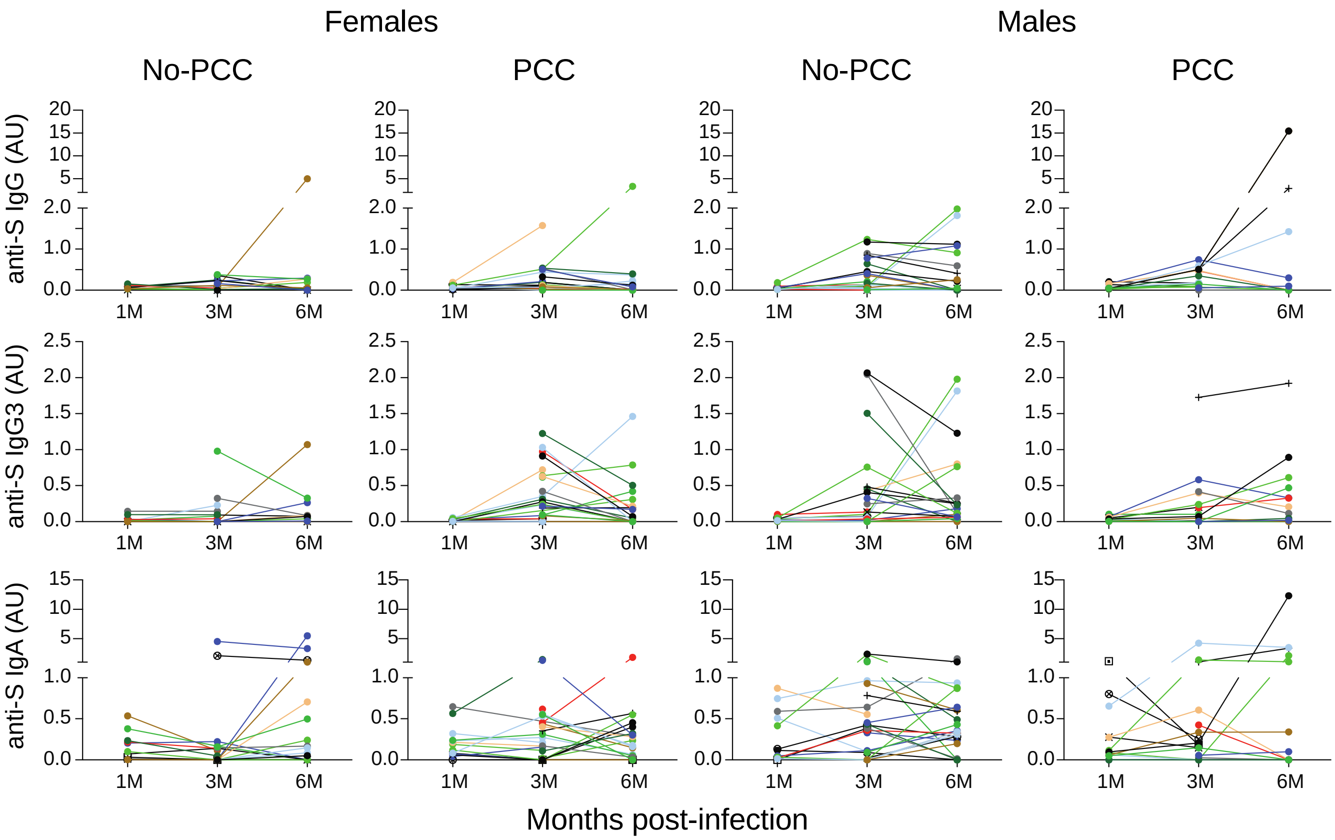


**Supplementary Fig 9. Evolution of anti-spike IgG, IgG3 and IgA antibody response from 1 month to 6 months in females and males with or without PCC.**

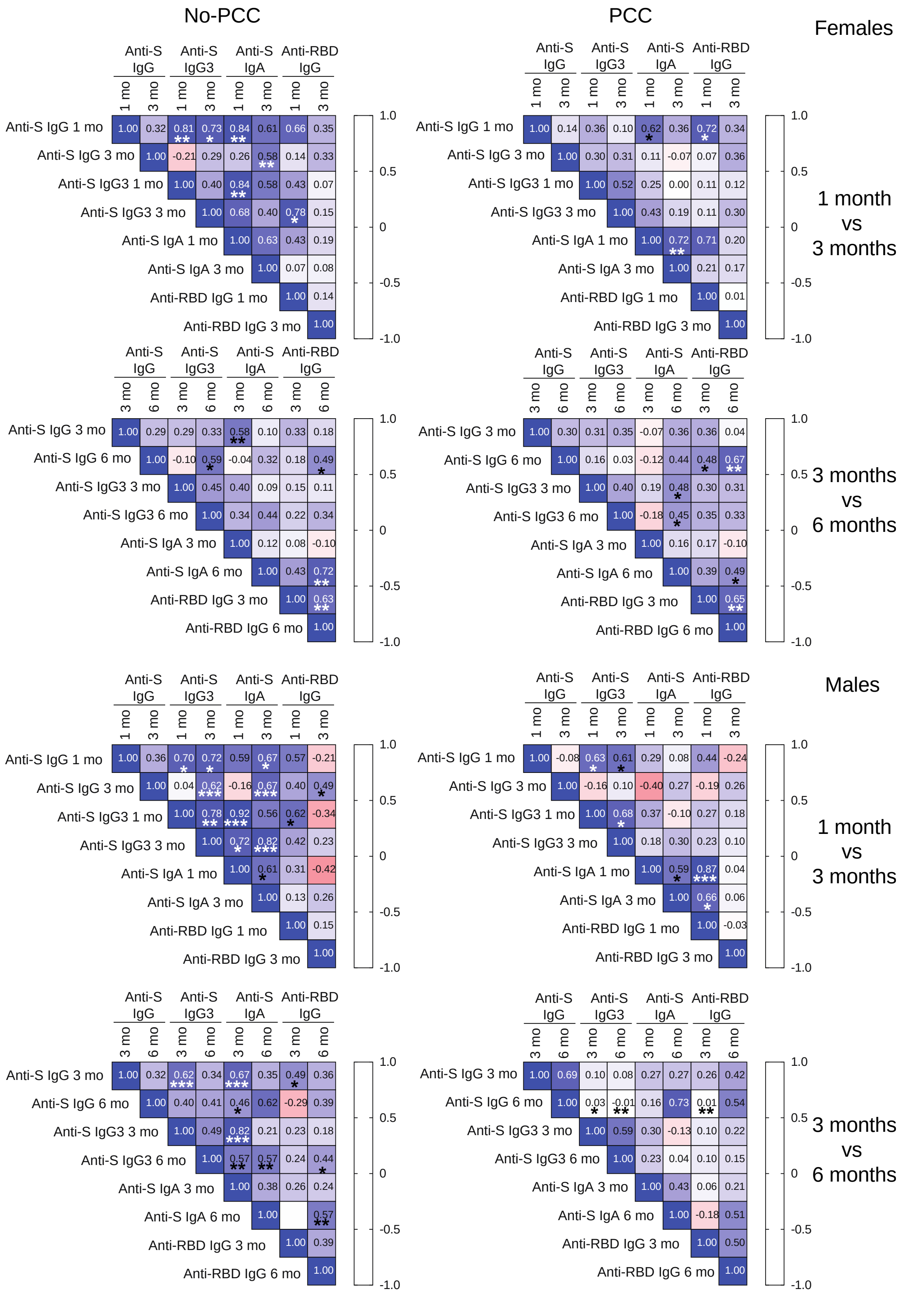


**Supplementary Fig. 10. Correlation matrix for antibody responses between 1- and 3- months and 3- and 6-months post infection in females and males with and without PCC.** RBD refers to RBD-

D614G. The numbers in the squares indicate the Spearman coefficient value. The *p* values are indicated in the figure as asterisks ( * $p<0.05$, ** $p<0.01$, *** $p<0.001$) and are given in **Supplementary Table S7.**

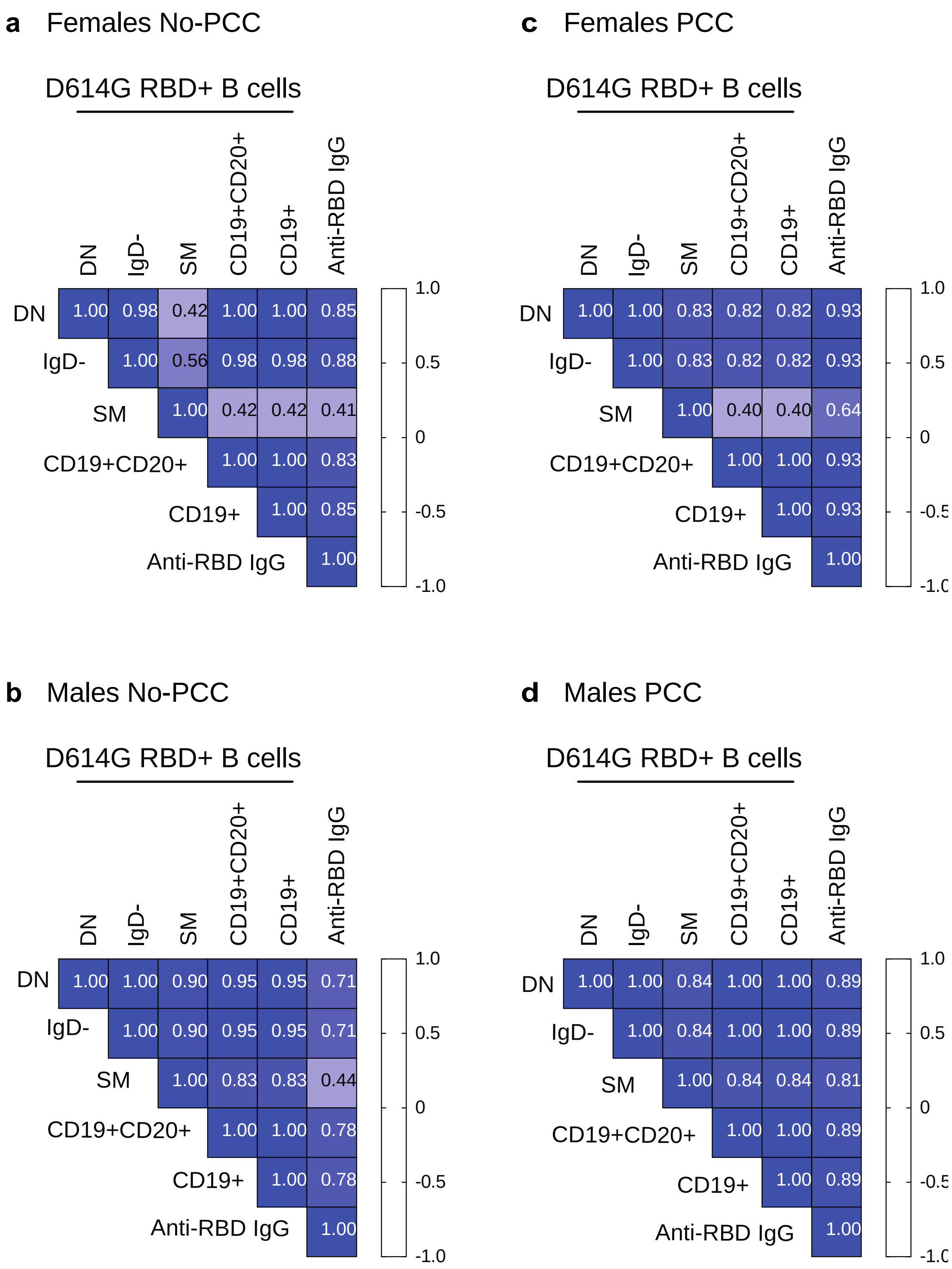

**Supplementary Fig. S11. Correlation matrix for anti-RBD responses at 12 in all the samples irrespective of their vaccination status.** The numbers in the squares indicate the Spearman coefficient value. The *p* values given in **Supplementary Table S8** are not significant**.**
